# Prevalence and evolution of intimate partner violence before and during pregnancy: a cross-sectional study

**DOI:** 10.1186/1471-2393-14-294

**Published:** 2014-08-28

**Authors:** An-Sofie Van Parys, Ellen Deschepper, Kristien Michielsen, Marleen Temmerman, Hans Verstraelen

**Affiliations:** Faculty of Medicine and Health Sciences, Department of Obstetrics and Gynaecology, Ghent University, International Centre for Reproductive Health, Belgium, De Pintelaan 185, UZP 114, 9000 Gent, Belgium; Faculty of Medicine and Health Sciences, Department of Public Health, Biostatistics Unit, Ghent University, De Pintelaan 185, 4K3, 9000 Gent, Belgium

**Keywords:** Intimate partner violence, Abuse, Pregnancy, Prevalence, Evolution, Pattern

## Abstract

**Background:**

Intimate partner violence (IPV) before and during pregnancy is associated with a broad range of adverse health outcomes. Describing the extent and the evolution of IPV is a crucial step in developing interventions to reduce the health impact of IPV.

The objectives are to study the prevalence of psychological abuse, as well as physical & sexual violence, and to provide insight into the evolution of IPV 12 months before and during pregnancy.

**Methods:**

Between June 2010 and October 2012, a cross-sectional study was conducted in 11 antenatal care clinics in Belgium. Consenting pregnant women were asked to complete a questionnaire (available in Dutch, French and English) in a separate room. Ethical clearance was obtained in all participating hospitals.

**Results:**

The overall percentage of IPV was 14.3% (95% CI: 12.7 - 16.0) 12 months before pregnancy and 10.6% (95% CI: 9.2 - 12.1) during pregnancy. Physical partner violence before as well as during pregnancy was reported by 2.5% (95% CI: 1.7 - 3.3) of the respondents (n = 1894), sexual violence by 0.9% (95% CI 0.5 - 1.4), and psychological abuse by 14.9% (95% CI: 13.3 - 16.7). Risk factors identified for IPV were being single or divorced, having a low level of education, and choosing another language than Dutch to fill out the questionnaire. The adjusted analysis showed that physical partner violence (aOR 0.35, 95% CI: 0.22 - 0.56) and psychological partner abuse (aOR 0.7, 95% CI: 0.63 - 0.79) were significantly lower during pregnancy compared to the period of 12 months before pregnancy. The difference between both time periods is greater for physical partner violence (65%) compared to psychological partner abuse (30%). The analysis of the frequency data showed a similarly significant evolution for physical partner violence and psychological partner abuse, but not for sexual violence.

**Conclusion:**

The IPV prevalence rates in our study are slightly lower than what can be found in other Western studies, but even so IPV is to be considered a prevalent problem before and during pregnancy. We found evidence, however, that physical partner violence and psychological partner abuse are significantly lower during pregnancy.

**Electronic supplementary material:**

The online version of this article (doi:10.1186/1471-2393-14-294) contains supplementary material, which is available to authorized users.

## Background

It is increasingly being recognized that intimate partner violence (IPV) is a global health problem with serious clinical and societal implications [1]. IPV is defined as any behaviour within a present or former intimate relationship that leads to physical, sexual or psychological harm, including acts of physical aggression, sexual coercion, psychological abuse and controlling behaviour patterns [2]. IPV is also known as domestic/family violence, spouse/partner abuse/assault, battering, violence against women or gender-based violence [3–5]. Based on the Centre for Disease Controle definition of IPV [6], we have chosen to consistently use the term ‘violence’ for physical and sexual types of violence, and ‘abuse’ for psychological types. The word ‘abuse’ clearly refers to a broader range of behaviours than the word ‘violence’, which is often associated with severe forms of violent behaviour.

Pregnancy and childbirth are major milestones in the lives of most couples and their families. The transition to parenthood brings joy but also new challenges to couple relationships [7, 8]. Pregnancy may be a stressful time because of changes in physical, emotional, social and economic requirements and needs in both (future) parents. Research on this matter [9–11] demonstrates that individual and dyadic coping strategies tend to decrease under stress, leading to an increased risk of physical and psychological aggression. This vulnerable period, however, is not limited to the time between conception and birth. Researchers have clearly demonstrated that risk factors for IPV associated with pregnancy encompass the time frame of one year before conception until one year after childbirth [11–15]. The mechanisms and determinants that influence the interaction between IPV and pregnancy, are not well-known. Four different patterns of (partner) violence around the time of pregnancy have been identified in the literature: (a) commencement of violence (no violence before pregnancy, but violence during pregnancy), (b) continuation of violence (violence both before and during pregnancy, either remaining unchanged or increasing/decreasing), (c) termination of violence (violence before pregnancy, but no violence during pregnancy), and (d) no violence (either before or during pregnancy). These patterns remain an important pathway to research because little is understood about how partner violence may change throughout a woman’s pregnancy, what factors contribute to the varying patterns, and why pregnancy appears to be a protective period for some women while it is a period of increased risk for others [9, 12, 16].

In the last 30 years, in the medical and psycho-social field more than one hundred studies on violence during pregnancy have been published in the Western world. Recently, more evidence has been emerging from low and middle-income countries [17]. Despite this considerable amount of research, sound estimates of the prevalence of abuse and violence during the childbearing period are difficult to obtain [18]. Available estimates of IPV around the time of pregnancy vary between 3 and 30%. Although estimates within regions and countries are highly variable, the majority of studies show prevalence rates ranging from 3.9% to 8.7% [19]. A recent systematic review [12] of prevalence studies of violence during pregnancy, reported 0.9 - 30% physical violence, 1 – 3.9% sexual violence and 1.5 – 36% psychological abuse during pregnancy. James et al. [20] calculated a mean reported prevalence rate of domestic (partner) violence among pregnant women of 19.8% over 92 studies. In Belgium, 10 years ago Roelens and colleagues [21] found a prevalence of 2.4% with respect to physical and/or sexual partner violence 12 months preceding pregnancy and of 2.2% with respect to physical and/or sexual partner violence during pregnancy. The variation in prevalence rates is influenced by the considerable differences in definitions (e.g. physical and/or sexual and/or psychological violence/abuse, domestic violence vs. IPV), study populations (e.g. small health-care based samples vs. population-based samples), the mode of inquiry (e.g. face-to-face interview vs. self-administered questionnaire), type of questions (e.g. general questions vs. specific behaviour) and the timing of inquiry (e.g. single measurement early in pregnancy vs. multiple measurements throughout the whole pregnancy). In other words, myriad study design features have influenced the prevalence rates reported, making comparison across studies a true challenge [12, 15, 18, 22, 23].

Over the last decades, research has generated growing evidence that IPV is linked to a broad range of adverse health outcomes and risk behaviour. A cohort study of Australian women aged 18–44 years estimated that intimate partner violence was responsible for 7.9% of the overall burden of disease, which was larger than other risk factors such as blood pressure, tobacco, and obesity [24]. IPV is therefore considered as an important contributor to the global burden of disease for women of reproductive age.

There is a large consensus among researchers and caregivers that the perinatal-care context is an ideal ‘window of opportunity’ to identify and address IPV, for it is often the only moment in the lives of many couples when there is (regular) contact with health care providers [19, 25]. Knowing the precise national prevalence of IPV is a first step in helping to inform the development and implementation of interventions to prevent and treat sequelae [19].

The objective of this paper is to determine the prevalence of physical, sexual (partner) violence and psychological (partner) abuse 12 months before and/or during pregnancy in Flanders, Belgium. First, this paper will explore the prevalence in subgroups offering rich information about the type of violence (physical, sexual, psychological), the perpetrator, the timing and the association with socio-demographic characteristics. Second, this paper will elaborate on the evolution of IPV 12 months before and/or during pregnancy.

## Methods

### Setting/study population

We conducted a multi-centre cross-sectional study in Flanders, the Northern part of Belgium. The Belgian perinatal health-care system is based on the medical model [26] and is generally considered highly accessible, with women choosing their own health care provider(s). Obstetricians/gynaecologists function as primary perinatal health-care providers and the majority of the care is hospital-based. Screening or systematic inquiry for IPV is not part of routine perinatal care.

This study was part of a RCT (Randomized Controlled Trial) that aims to assess the impact of an intervention on psychosocial health, IPV, safety and help-seeking behaviour. We recruited in 11 antenatal care clinics, in order to obtain a representative sample of the general obstetric population. The convenience sample of hospitals was geographically spread over Flanders, and had a balanced mix of rural and urban settings, as well as small and large hospitals, providing services to economically and ethnically diverse populations.

From June 2010 until October 2012, pregnant women consecutively seeking antenatal care were invited to participate in the study if they were at least 18 years old and able to fill out a Dutch, French or English questionnaire. The study was limited to one questionnaire per woman and we did not impose limits on the gestational age. The midwife or secretary introduced the study as a survey on difficult moments and feelings during pregnancy and briefly explained the procedure. Consenting women were handed a questionnaire, including an informed-consent form, which was filled in in a separate room without any accompanying person present. If the woman was unable to fill in in private, she was excluded from the study for safety reasons. The overall response rate was 76.7%.

**Figure 1:**
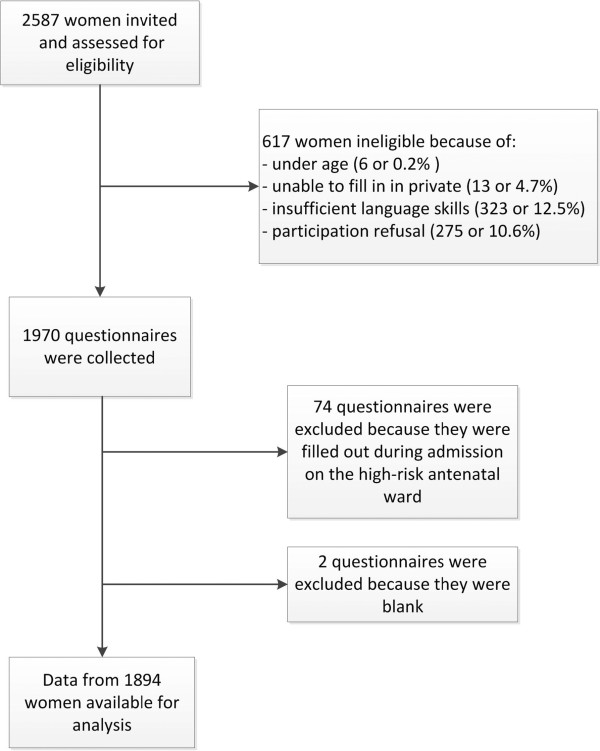
**Flow diagram recruitment.**

The study was approved by the Ethics Committee of Ghent University and local ethical clearance from all 11 participating hospitals was obtained (Ethisch Comité Middelheim Ziekenhuis Netwerk Antwerpen, Ethisch Comité Universitair Ziekenhuis Antwerpen, Ethisch Comité Onze Lieve Vrouw Ziekenhuis Aalst, Ethisch Comité Gasthuis Zusters Ziekenhuis St Augustinus Antwerpen, Ethisch Comité Algemeen Ziekenhuis Sint Jan Brugge, Ethisch Comité Algemeen Ziekenhuis Jan Palfijn Gent, Ethisch Comité Onze Lieve Vrouw van Lourdes Ziekenhuis Waregem, Ethisch Comité Universitair Ziekenhuis Gent, Ethisch Comité Algemeen Ziekenhuis Groeninge Kortrijk, Ethisch Comité Virga Jesse Ziekenhuis Hasselt, Ethisch Comité Ziekenhuis Oost-Limburg Genk) (Belgian registration number 67020108164). The trial was registered at http://www.clinicaltrials.gov, identifier NCT01158690 (http://clinicaltrial.gov/ct2/show/NCT01158690?term=violence+and+pregnancy&rank=1).Figure 1 gives an overview of the study sample collection.

The questionnaires were scanned and processed using the software Remark Office OMR version 7 and exported to SPSS version 21. The data file was rigorously checked by two researchers for data entry errors. To check the quality of the scanning process, a random sample of 100 questionnaires was controlled by hand, yielding an error rate of 1%.

### Questionnaire/measures

The self-administered questionnaire (see Additional file 1) consists of four main parts: socio-demographics, psycho-social health, violence, and satisfaction with care. This paper focuses on the prevalence and evolution of IPV. The questionnaire was available in Dutch, French and English and was based on a thorough translation and back translation of the original instruments.

Physical and sexual violence was measured by using the Abuse Assessment Screen (AAS) [27], which was adapted in close consultation with one of the authors (Prof. dr. Judith McFarlane). The following questions were used:

1. Have you ever been emotionally or physically abused by your partner or someone important to you?
2. During the 12 months prior to your pregnancy/since you became pregnant: were you hit, slapped, kicked or otherwise physically hurt by someone?
3. During the 12 months prior to your pregnancy/since you became pregnant: did anyone force you to have sexual activities?

Response alternatives were yes/no. Questions 2 & 3 also included explicit questions about the perpetrator (spouse, ex-spouse, partner, ex-partner, family member, stranger, other) and frequency (rarely, occasionally, often, very often). For the pregnancy period we explored the evolution through the following question ‘In terms of its severity and/or frequency, has this behaviour increased, decreased, or remained unchanged’. A positive answer to question 1 was defined as lifetime abuse. A positive, negative or missing answer to questions 2 & 3 in combination with one or more positive answers to the sub questions on perpetrator, frequency and evolution was defined as physical and sexual violence respectively. The value was considered as missing if it was missing for all questions and sub questions and this never exceeded 4% (n = 75). Women indicating a spouse, ex-spouse, partner, ex-partner as perpetrator were classified as experiencing partner violence. After the questions on physical and sexual violence we included the following question: ‘Are you afraid of your partner or anyone you listed above?’ to be able to compare the detection rates of the different screening questions.

To measure psychological abuse, we used an adapted version of the WHO-questionnaire [3]. The following questions were used: When you think about your current or last partner, did he/she in the 12 months prior to your pregnancy/since you became pregnant:

1. try to restrict your contact with male/female friends and/or family?
2. insist on knowing where you are at all times?
3. ignore you and treat you indifferently?
4. insult you, criticize you, or react in a despising manner to what you do or say?
5. belittle or humiliate you in front of other people?
6. do things to scare or intimidate you on purpose? [e.g. smashing things, threatening to kill you or to commit suicide]
7. threaten to hurt you or someone you care about?

We assessed the evolution of violence during the pregnancy period (increased, decreased, or unchanged) if a minimum of one question was answered with at least ‘rarely’. Psychological abuse by a non-partner (family member, stranger, other) was measured by ‘Did someone else than your current or last partner behave in more than one of the above-mentioned ways?’ with sub questions on who and when.

Contrary to the situation for physical and sexual violence, there is currently a lack of agreement on standard measures for psychological (partner) abuse/violence and the threshold at which behaviour crosses the line of becoming psychological abuse/violence [1]. In an effort to tackle this problem, we composed a scale based on the 7 questions above with response alternatives never (=0), rarely (=1), occasionally (=2), often (=3) and very often (=4). Based on the limited available literature [1, 3, 16, 28–34] we decided to use a cut-off value of 4/28 as a threshold for psychological abuse, and hence a score of 3 or lower was not considered psychological abuse. Our scale had a good internal consistency, with a Cronbach’s α value of 0.85 for 12 months before pregnancy and of 0.83 during pregnancy. The proportion of missing values for the questions on psychological abuse was 10.2% (n = 193).

In an attempt to overcome the methodological challenges associated with comparing a measurement period of 12 months before pregnancy with the pregnancy period itself, which was on average 23.9 weeks, we created a frequency variable for partner violence including the answering options of ‘never, rarely, occasionally, often & very often’. This variable is built up in a similar way as the above partner violence variables with the additional condition of a valid value on the frequency question. Since the answering categories contain a certain time dimension and the question was repeated for both time periods, the women were asked to make a subjective comparison of the evolution of the IPV. Despite the fact that we cannot exclude the impact of the time dimension, we believe that this frequency variable yields the best possible approximation of a ‘true’ evolution.

### Data-analysis

A descriptive analysis of the socio-demographic variables, violence, perpetrator, frequency and evolution data was performed. Prevalences of physical and sexual violence and psychological abuse 12 months prior to pregnancy and during pregnancy are reported together with their 95% Wilson Score confidence intervals. The intervals were obtained in R (version 3.0.1), using the ‘scoreci’ function in the R-library PropCIs_0.2-0 [35].

The McNemar test was used to assess the significance level of the difference between two paired proportions of IPV (12 months before vs. during pregnancy). P values below 0.05 were considered to be statistically significant.

For each type of violence a Generalized Estimating Equation (GEE) analysis was used to investigate the differences in the odds of violence for both time periods and perpetrators. The analyses were adjusted for age, gestational age, language in which the questionnaire was filled out, civil/marital status and education. Logistic regression analysis was used to assess socio-demographic risk factors for IPV. Odds ratios (95% confidence intervals) were used to determine the association of the type of violence with the time periods and socio-demographic factors. For the analysis of the evolution of IPV based on the frequency variable, we assessed the statistical significance using marginal homogeneity tests. Statistical analyses were performed in IBM SPSS statistics (version 21).

This research adhered to the STROBE guidelines for cross-sectional studies as outlined in http://www.strobe-statement.org/fileadmin/Strobe/uploads/checklists/STROBE_checklist_v4_cross-sectional.pdf (checklist added as Additional file 2).

## Results

### Socio-demographic data

The mean age of our sample was 28.9 years (SD 4.5) and the median gestational age was 21 weeks (P25 = 19 & P75 = 30). The large majority (95%) of the women were married or living together, 5% was divorced, separated or single. 62.1% completed higher education and 37.8% did not. Most women chose to fill out the questionnaire in Dutch (97.5%), 0.9% in French and 1.6% in English. More details are presented in Table 1.

**Table 1:** **Socio-demographic characteristics of sample (n = 1894)**

Characteristics	Frequency (n)	%
Age (n = 1842)		
15-19 (minimum age 18)	31	1.7
20-24	262	14.2
25-29	742	40.3
30-34	626	34.0
35-39	149	8.1
40-44	31	1.7
45-49	1	0.1
Civil/marital status (n = 1880)		
Married	928	49.4
Living together	857	45.6
Divorced or separated	13	0.7
Single	82	4.4
Education (n = 1878)		
None	34	1.8
Primary education	76	4.0
Secondary education	601	32.0
Non-university higher education	800	42.6
University higher education	367	19.5
Language questionnaire (n = 1894)		
Dutch	1846	97.5
French	17	0.9
English	31	1.6

### Overall prevalence

The prevalence of abuse committed by a partner or a significant other during lifetime was 12.1% (n = 225). Twenty-two women (or 1.2% of the total sample) reported being afraid of their partner or another perpetrator at the time of filling out the questionnaire.

The detailed prevalence rates of physical and sexual violence and psychological abuse are presented in Table 2.

**Table 2:** Prevalence of physical and sexual violence and psychological abuse in the 12 months before pregnancy and during pregnancy (n = 1894)* with 95% **Wilson Score confidence intervals**

	Partner % (n)	Non-partner % (n)	Total % (n)*
	95% CI	95% CI	95% CI
Physical violence in the 12 months before pregnancy	2.3 (42) (1.7 – 3.0)	1.6 (30) (1.1 – 2.3)	4.2 (78) (3.4 – 5.2)
Physical violence during pregnancy	0.8 (14) (0.5 – 1.3)	1.1 (20) (0.7 – 1.6)	2.4 (44) (1.8 – 3.2)
**Total physical violence**	2.5 (45) (1.8 – 3.3)	2.0 (38) (1.5 – 2.8)	4.8 (88) (3.9 – 5.8)
Sexual violence in the 12 months before pregnancy	0.6 (11) (0.3 – 1.1)	0.1 (1) (0.3 – 0.5)	0.8 (14) (0.5 – 1.3)
Sexual violence during pregnancy	0.5 (10) (0.3 – 1.0)	0.2 (3) (0.05 – 0.5)	1.1 (20) (0.7 – 1.7)
**Total sexual violence**	0.9 (16) (0.5 – 1.4)	0.2 (3) (0.05 – 0.5)	1.4 (26) (1.0 – 2.1)
Psychological abuse in the 12 months before pregnancy	13.6 (236) (12.1 – 15.3)	3.3 (59) (2.6 – 4.2)	16.3 (278) (14.7 – 18.2)
Psychological abuse during pregnancy	10.1 (175) (8.8 – 11.6)	3.1 (55) (2.4 – 4.0)	12.8 (218) (11.3 – 14.5)
**Total psychological abuse**	14.9 (257) (13.3 – 16.7)	4.6 (83) (3.7 – 5.7)	18.5 (316) (16.8 – 20.5)
**Total violence all periods**	15.8 (270) (14.2 – 17.7)	6.3 (114) (5.3 – 7.5)	20.4 (347) (18.6 – 22.4)

*The total percentages reflect violence by a partner and/or non-partner (family member, stranger, other). Since one respondent could tick off several types of violence, the total percentages do not add up to 100. The total percentages also include women responding positive to one of the violence questions, but where the specific perpetrator was unknown.

IPV in both periods (12 months before and/or during pregnancy) was 15.8% (n = 270), non-partner violence in both periods was 6.3% (n = 114) and overall violence in both periods was 20.4% (n = 347). Physical violence in both periods by any perpetrator was 4.8% (n = 88), sexual violence in both periods by any perpetrator was 1.4% (n = 26) and psychological abuse in both periods by any perpetrator was 18.5% (n = 316).

### Perpetrator of IPV before and/or during pregnancy

Physical **partner** violence 12 months before and/or during pregnancy was reported by 2.5% (n = 45) of the women, sexual partner violence by 0.9% (n = 16), and psychological partner abuse by 14.9% (n = 257) of our sample. Physical violence by a **non-partner** (family member, stranger, other) 12 months before and/or during pregnancy was 2.0% (n = 38), sexual violence 0.2% (n = 3) and psychological abuse 4.6% (n = 83).

The descriptive results of this study show that 58.3% (n = 42) of the known perpetrators of physical violence 12 months before pregnancy were identified as (ex)partners, while 41.7% (n = 30) were non-partners. This proportion is reversed during pregnancy, with 40% (n = 14) partners and 60% (n = 21) non-partners. The known perpetrators of sexual violence 12 months before pregnancy consisted of 91.7% (n = 11) (ex)partners and 8.3% (n = 1) non-partners. During pregnancy 76.9% (n = 10) of identified sexual violence perpetrators were (ex)partners and 23.1% (n = 3) non-partners. The known perpetrators of psychological abuse 12 months before pregnancy consist of 84.8% (n = 236) (ex)partners and 21.2% (n = 59) non-partners. This proportion remains similar during pregnancy and is 80.3% (n = 175) and 25.2% (n = 55).

### Comparison of prevalence before and during pregnancy

The total incidence percentage of IPV **12 months before pregnancy** was 14.3% (n = 246) and the total incidence percentage of IPV during pregnancy was 10.6% (n = 181), based on 1684 women who reported IPV for both periods. IPV **during pregnancy** is significantly lower statistically (*P <* 0.001) than it is during the 12 months before pregnancy.

IPV **only** 12 months before pregnancy but not during pregnancy, was reported by 4.5% of the total sample and this is 30.4% (76/250) of the total IPV. IPV only during pregnancy but not in the 12 months before pregnancy, was reported by 1.0% of the total sample and this is 6.8% (17/250) of the total IPV.

### Combination of violence types

Of all the women who reported IPV 12 months before pregnancy, the majority (85.2% or n = 201) indicated only **one type** (physical or sexual or psychological) of partner violence, while 14.8% (n = 34) reported **2 or 3 types** of violence. The proportion during pregnancy was 91.4% (n = 149) of the respondents reporting one type of violence and 8.6% (n = 14) 2 or 3 types. Furthermore, women reported significantly (*P* < 0.001) fewer combinations of several types of violence during pregnancy as compared to the situation in the 12 months before pregnancy, based on the 1669 women who reported IPV for both periods.

### Evolution of violence

The results from the unadjusted GEE analysis show that physical partner violence during pregnancy (0.8%, 95% CI: 0.5 – 1.3) is statistically significantly (*P* < 0.001) lower than physical partner violence 12 months before pregnancy (2.3%, 95% CI: 1.7 – 3.0). The difference in physical violence by a non-partner over both periods marginally missed significance [*P* = 0.050, 1.6% (95% CI: 1.1 – 2.3) vs. 1.1% (95% CI: 0.7 – 1.6)]. Furthermore, the evolution is significantly stronger (*P* = 0.036) for physical partner violence than for non-partner violence.

Sexual partner violence during pregnancy (0.5%, 95% CI: 0.3-1) is not statistically significantly lower (*P* = 0.772) than sexual partner violence 12 months before pregnancy (0.6%, 95% CI: 0.3-1.1). Sexual violence by a non-partner did also not change significantly [*P* = 0.157, 0.2% (95% CI: 0.1 – 0.5) vs. 0.05% (95% CI: 0.01 – 0.4)]. No significant difference in the evolution of sexual violence between partners and non-partners could be found (*P* = 0.173).

Psychological partner abuse during pregnancy (10.1%, 95% CI: 8.8 – 11.6) is statistically significantly (*P* < 0.001) lower than psychological partner abuse 12 months before pregnancy (13.6%, 95% CI: 12.1 – 15.3). Psychological abuse by a non-partner did not change significantly [*P* = 0.433, 3.1% (95% CI: 2.4 – 4.0) vs. 3.3% (95% CI: 2.6 – 4.2)]. The evolution of psychological partner abuse is significantly stronger (*P* = 0.014) than the decrease in violence by a non-partner.

The estimated odds of physical partner violence (OR 0.33, 95% CI: 0.21 – 0.54) during pregnancy decreased by 66.7% and psychological partner abuse (OR 0.71, 95% CI: 0.64 – 0.80) by 28.7% compared to the situation in the 12 months before pregnancy (more details are available in Table 3).

**Figure 2:**
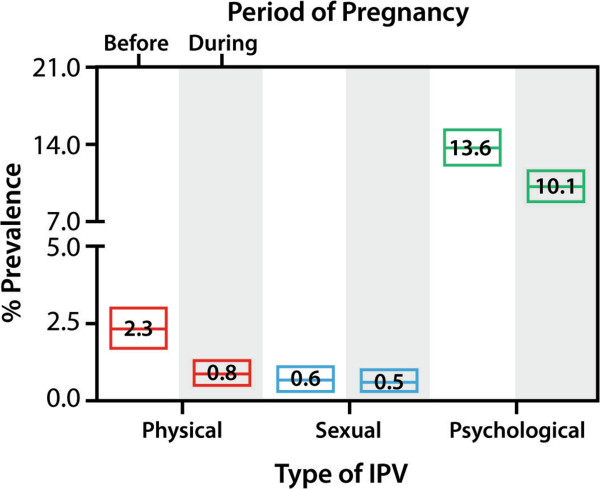
**Comparison of prevalence rates 12 in the months before pregnancy with those during pregnancy (median gestational age 21 weeks).** The upper side of the box refers to upper limit of 95% CI, lower side to lower limit of 95% CI of the prevalence. The middle line in the box indicates the prevalence rate.

Figure 2 provides a clear illustration of the evolution of the different types of IPV in the period from 12 months before pregnancy to the period during pregnancy (median gestational age 21 weeks).

**Table 3:** **Overview of odds and adjusted odds of violence for both time periods and perpetrators**

	OR 95% CI (GEE 1)	***P***-value	aOR 95% CI (GEE 2)**	***P***-value
**Physical**				
Partner violence during pregnancy*	0.33 (0.21 – 0.54)	< 0.001	0.35 (0.22 – 0.56)	< 0.001
Non-partner violence during pregnancy	0.66 (0.44 – 1.00)	0.052	0.70 (0.45 – 1.08)	0.104
**Sexual**				
Partner violence during pregnancy	0.91 (0.49 – 1.70)	0.772	0.95 (0.48 – 1.90)	0.894
Non-partner violence during pregnancy	3.01 (0.61 – 14.93)	0.177	3.11 (0.62 – 15.74)	0.170
**Psychological**				
Partner violence during pregnancy	0.71 (0.64 – 0.80)	<0.001	0.70 (0.63 – 0.79)	<0.001
Non-partner violence during pregnancy	0.93 (0.78 – 1.12)	0.433	0.93 (0.77 – 1.12)	0.432

*Reference category 12 months before pregnancy.

**Adjusted for language of the questionnaire, civil/marital status, education and age.

The results of the binary logistic regression analysis for IPV (in both periods), are shown in Table 4. This analysis demonstrates that the language used to fill out the questionnaire, civil/marital status and education have a significant impact on the prevalence of IPV in both periods, while age does not. In the bivariate analysis, age was significantly correlated to IPV, but when age was added to the model, the correlation was filtered out by the other socio-demographic factors.

**Table 4:** **Overview of socio-demographic risk factors for IPV**

	aOR IPV (both periods)	95% CI	***P***-value
Language questionnaire			
Dutch (ref)	1		
French	4.68	1.36 – 16.10	0.014
English	7.44	3.07 – 18.1	< 0.001
Civil/marital status			
Married/cohabiting (ref)	1		
Divorced/single	4.48	2.55 – 7.88	< 0.001
Education			
Higher/university education (ref)	1		
No/primary education	6.74	3.94 – 11.55	< 0.001
Secondary education	2.64	1.94 – 3.358	< 0.001
Age	0.99	0.96 – 1.02	0.475

When a woman reported lifetime abuse, we found an aOR of 5.37 (95% CI: 4.03 – 7.15) for IPV in both periods.

In a second GEE analysis, we investigated the differences in adjusted odds of partner and non-partner violence over both time periods (see Table 3). After correction for age, language used to fill out the questionnaire, civil/marital status and education, the aOR for physical partner violence during pregnancy (0.35, 95% CI: 0.22 – 0.56) turned out to be significantly (*P* < 0.001) lower than in the period of 12 months before pregnancy. The aOR for physical non-partner violence during pregnancy (0.7, 95% CI: 0.45 – 1.08) is not significantly (*P* = 0.104) lower than that of the period 12 months before pregnancy. The adjusted odds for physical partner violence during pregnancy decrease with 65% as compared to those in the 12 months before pregnancy, whereas physical non-partner violence decreased with 30%. The evolution of physical partner violence was statistically significantly (*P* = 0.043) stronger than the evolution in physical non-partner violence.

The aOR for sexual partner violence during pregnancy (1.06, 95% CI: 0.53 – 2.13) is not significantly (*P* = 0.869) lower than during the 12 months before pregnancy. The aOR for sexual non-partner violence during pregnancy (2.05 95% CI: 0.50 – 8.43) is not statistically significantly (*P* = 0.318) lower than that of the 12 months before pregnancy. No statistical significant (*P =* 0.413) difference in evolution of sexual violence between partners and non-partners could be found.

The aOR for psychological partner abuse during pregnancy (0.70, 95% CI: 0.63 – 0.79) is significantly (*P* < 0.001) lower than that in the 12 months before pregnancy. The aOR for psychological non-partner abuse during pregnancy (0.93, 95% CI: 0.77 – 1.12) is not statistically significantly (*P* = 0.432) lower than that of the 12 months before pregnancy. The odds for psychological partner abuse during pregnancy decrease with 30% compared to those for the period of 12 months before pregnancy. Psychological non-partner abuse decreased with 7% but this was not statistically significant. The evolution of psychological partner abuse is significantly (*P* = 0.014) stronger than the evolution in psychological non-partner abuse. We observed a larger decrease in physical partner violence as compared to that in psychological partner abuse.

When a woman reported IPV in the 12 months before pregnancy, she had an aOR of 165.39 (95% CI: 90.52 – 302.19) for IPV during pregnancy. The likelihood of reporting physical partner violence is 30.7% (95% CI: 19.71 – 44.33). For sexual partner violence it is 54% (95% CI: 35.6 – 71.32), and for psychological abuse 68.74% (95% CI: 65.8 – 71.54) in cases where the different types of partner violence were reported in the 12 months before pregnancy.

Subsequently, we explored the impact of gestational age on the evolution of IPV over both time periods. We found that gestational age is significantly associated with IPV during pregnancy and has an aOR of 1.03 (95% CI: 1.01 – 1.05).

As already pointed out in the Methods section, the outcome variables used in the above analysis include two different time periods i.e. 12 months before pregnancy and the period during pregnancy with a median gestational age of 21 weeks. In an effort to address this methodological challenge, we created a frequency variable for partner violence. Since the answering categories of this variable contain a certain time dimension and the question was repeated for both time periods, the women were asked to make a subjective comparison of the evolution of the IPV. Despite the fact that we cannot exclude the impact of the time dimension, we believe that this frequency variable yield the best possible approximation of a ‘true’ evolution.

The results of the bivariate analysis of the IPV frequency variable, based on the marginal homogeneity test, showed that the frequency of physical partner violence during pregnancy is significantly lower than the frequency of physical partner violence in the period 12 months before pregnancy (*P* < 0.001). The frequency of sexual partner violence during pregnancy is not statistically significantly different than the frequency of sexual partner violence 12 months before pregnancy (*P* = 0.537). The frequency of 6 out of 7 sub questions of psychological partner abuse during pregnancy is statistically significantly lower than those for 12 months before pregnancy (*P*-values between 0.002 and < 0.001). There was no significant change found (P = 0.091) for the sub question ‘Did your (ex)partner threaten to hurt you or someone you care about’.

Finally, in addition to the analysis above, we explored in a third GEE whether physical and sexual partner violence and psychological partner abuse increased, decreased, or remained unchanged during the pregnancy itself. We found no significant differences in the evolution of IPV (*P* = 0.19) during the pregnancy period.

## Discussion

The results of this study indicate that violence is a prevalent problem around the time of pregnancy in Flanders, Belgium. One fifth (20.4%) of the women report some form of IPV in the 12 months before and/or during pregnancy. (Ex-)partners account for the largest share (77.8%) of all violence reported and family members, strangers or others for 22.2%. Psychological abuse is the type IPV of that is reported most frequently.

Estimates of IPV around the time of pregnancy vary between 3 to 30%. Prevalence rates in African and Latin American countries are mainly situated at the high end of the continuum and the European and Asian countries are positioned at the lower end [19]. A recent systematic review [12] reported 0.9 - 30% physical violence, 1 – 3.9% sexual violence and 1.5 – 36% psychological abuse during pregnancy. We found 2.4% physical, 1.1 sexual and 12.8% psychological abuse during pregnancy. James et al. [20] reported a mean prevalence rate of domestic (partner) violence among pregnant women of 19.8% over 92 studies, whereas we found 10.5%. An earlier Belgian study by Roelens et al. [21] using a related assessment tool, reported 2.4% physical and/or sexual partner violence 12 months before pregnancy, whereas we found 2.6%. In contrast to the 2.2% physical and/or sexual partner violence during pregnancy in Roelens’ study [21], we only found 1.1%. Caution is recommended when interpreting and comparing results of different studies, as methodological differences and challenges are substantial (cf. introduction). Nonetheless, our results seem to be situated at the lower end of the different continuums found in other studies in Western antenatal clinical settings, and this was also confirmed by a European multi-country study in which we participated [36]. Possible hypotheses for this relatively low prevalence rate are that, compared to the general obstetric population in Flanders, our sample is more educated and only a minority chose to fill out the questionnaire in another language, which can both be considered as a proxy for a higher socio-economic status. Moreover, women were on average 24 weeks pregnant when they filled out the questionnaire, and it is not unthinkable that the violence starts after this gestational age. This may account for the lower recording rate of IPV during pregnancy in our study. Furthermore, the lower prevalence rates may also be attributed to the 25% of the women that opted not to participate in our study. A more optimistic hypothesis is that the women in our study actually experienced less violence compared to women in the above-mentioned studies. However, they might as well be more hesitant to disclose experiences of violence in a hospital-based survey or may not acknowledge certain behaviour as being transgressive.

The analysis of the prevalence and the frequency variable showed that physical partner violence and psychological partner abuse are significantly lower during pregnancy as compared to the 12-months period before pregnancy. Moreover, the evolution was stronger for physical partner abuse than for psychological partner abuse, suggesting that partners are generally less physically violent but not necessarily less psychologically abusive. We were not able to detect any evolution in sexual partner violence and this is probably linked to the small sample size. Similarly, other researchers have demonstrated that prevalence of violence during pregnancy is consistently lower than violence occurring before pregnancy, both in developed [14, 15, 37–50] and less developed nations [51–56]. Furthermore, we found that 6.3% of the total IPV occurred only during pregnancy (and not in the 12 months before), 28.1% of the women indicated that they only experienced IPV in the 12 months before (and not during) pregnancy. Likewise, Taillieu [12] found that 31 to 69% of the women indicate that IPV stopped during pregnancy. We do not dispose of concrete data explaining why IPV is lower during pregnancy, but one could hypothesize that pregnancy changes the social status of a woman [57] and that it increases social control and respect for the woman. Another hypothesis is that in Western societies a pregnant woman is seen as a receptacle for the vulnerable unborn child. Partners may realize that physical and sexual (not necessarily psychological) violence can harm the baby and therefore use less (physical) violence. We could also hypothesize that women feel more vulnerable during pregnancy and actually use more tactics to avoid violent escalations.

Though the prevalence of violence during pregnancy is found to be consistently lower than that of the pre-pregnancy period, 60% to 96% of the women who are abused during pregnancy also report having been abused in the past, suggesting that pregnancy violence represents a continuation of pre-existing violence for most pregnant victims [12]. This was confirmed by our finding that lifetime abuse and IPV in the 12 months before pregnancy are very strong predictors for IPV during pregnancy.

Similarly to what is found in most other studies [12, 19, 20, 58, 59], the results of our study illustrate that filling out the questionnaire in another language than Dutch, being divorced or single, and having a lower than secondary education (as proxies for low socio-economic status) are important risk factors which increase odds for the reporting of IPV substantially.

In the scientific literature, there is currently a debate on what question(s) should be used to identify (partner) violence. Some authors [60–64] have suggested that single question screening using ‘Are you afraid of…’, would be sufficient. When we compare the results of ‘Are you afraid of your partner or anyone you listed above?’ (1.2%, n = 22) with the results of a set of specific behavioural questions assessing IPV in the 12 months before and/or during pregnancy (20.4%, n = 347), it is clear that measuring violence by means of one general question detects much less violence. This finding has been confirmed by several other authors [12, 15, 18, 22, 23, 45]. An exploratory analysis of the 22 women that declared themselves to be afraid, revealed that these women had a lower socio-economic status, more psycho-social problems and higher violence prevalence rates. Using the ‘are you afraid’ question only seems to detect the tip of the iceberg and proves to be an inappropriate screening question in our study population.

The findings in this study are subject to several limitations. First, there is currently a lack of agreement on standard measures of emotional/psychological partner abuse/violence and the threshold at which acts can be considered being emotional/psychological abuse/violence [1]. The threshold we chose for psychological abuse was based on a thorough literature search and extensive discussions with experts in the field. Nevertheless, it remains an arbitrary choice that is open for discussion. Contrary to many other authors, we made the decision to actively contribute to the development of a standard measure and cut-off value for psychological partner abuse. Yet, we clearly acknowledge that our threshold is not beyond debate. Second, the comparison of prevalence rates of violence during pregnancy to those of the period of 12 months before pregnancy, should be interpreted with caution, since the period referring to pregnancy was on average half of the 12 months before pregnancy, which obviously reduces the chances of experiencing violence. In an attempt to overcome this methodological challenge, we analysed the frequency data as a best possible approximation of a ‘true’ evolution and found a similar evolution of IPV. However, since the questions on frequency were also linked to the time periods, we cannot exclude that the results are biased by the difference in measurement period. Moreover, we need to be careful about making statements about the evolution of violence since we do not dispose of data on the postpartum period and many researchers have shown that this is a period of increased violence [9, 11, 16, 58, 59, 65–67]. Third, we did not explicitly measure child abuse, financial distress or economic violence. These factors are known to be linked to violence and could have been used to adjust our analysis. Fourth, this part of the study only disposes of data on female victimization. Growing evidence shows that IPV not only involves female victims and male perpetrators, but that it is rather a matter where both partners/sexes contribute mutually [68, 69]. We therefore do not claim to create a representative image of reality where both partners play their roles.

## Conclusions

Our results demonstrate that in Flanders, Belgium, one out of five women experiences violence around the pregnancy period. Psychological abuse inflicted by (ex-)partners is the most frequent type of violence. Increasing evidence shows that the consequences of psychological abuse are as serious as those of physical and sexual violence. Although IPV seems to be lower during pregnancy, it remains a prevalent problem and not much is known about the specific reasons for this decrease. We call upon fellow researchers to breathe new life into the debate on the current methodological challenges associated with measuring IPV, especially the problem of comparing different measurement periods and the lack of a threshold for psychological abuse.

## Electronic supplementary material

Additional file 1: **Study questionnaire.** (PDF 2 MB)

Additional file 2: **STROBE Checklist.** (PDF 18 KB)
