# Supplementary material for: Prevalence and evolution of intimate partner violence before and during pregnancy: a cross-sectional study
Source: BMC Pregnancy Childbirth. 2014 Aug 28;14:294. doi: 10.1186/1471-2393-14-294 (PMC4159505; doi:10.1186/1471-2393-14-294)
Supplement: Supplementary file 1 — Additional file 1: Study questionnaire. (PDF 2 MB) [file 12884_2014_1167_MOESM1_ESM.pdf]

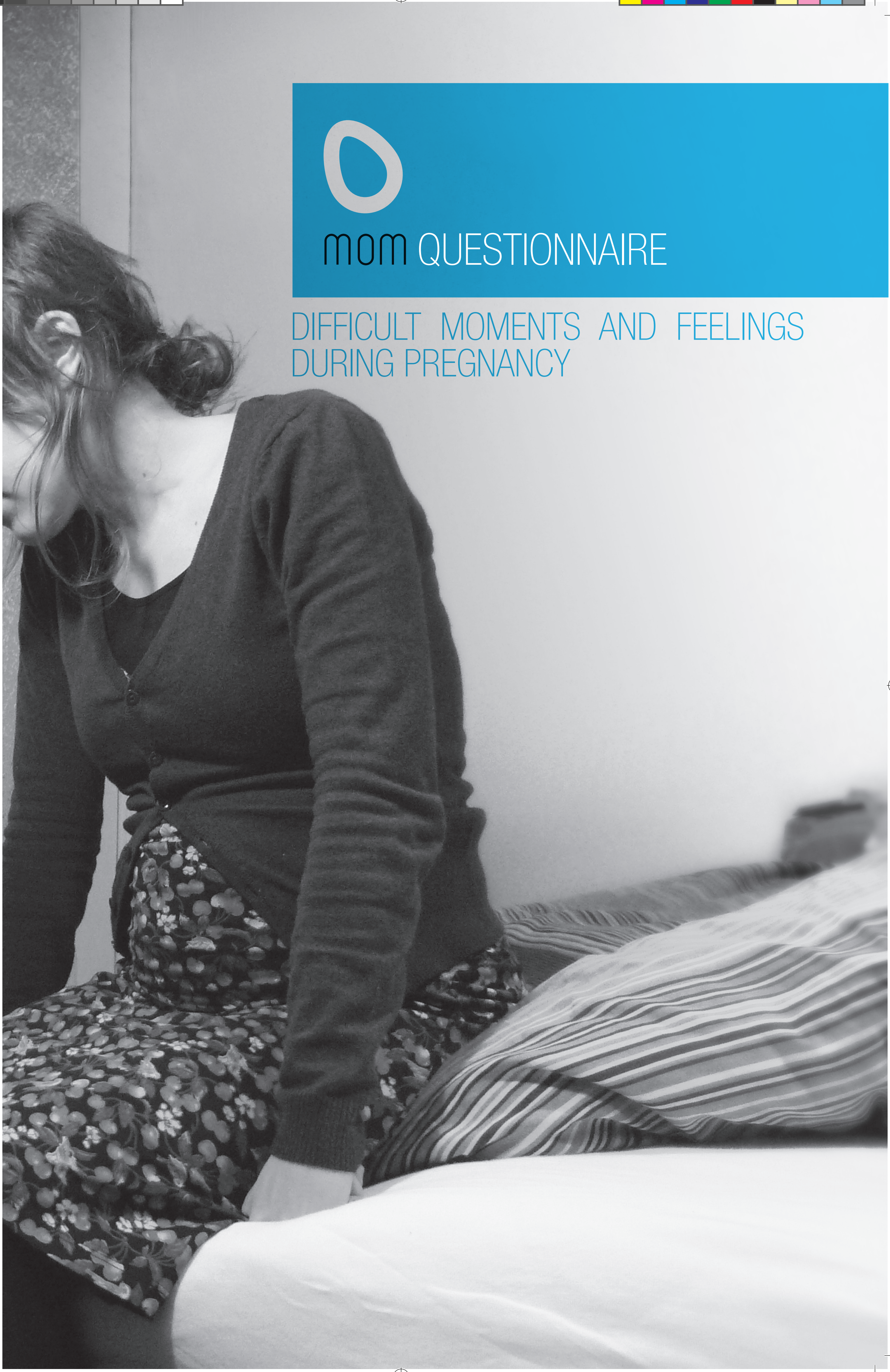

mom QUESTIONNAIRE

DIFFICULT MOMENTS AND FEELINGS DURING PREGNANCY

UNIVERSITEIT GENT | Women's Clinic, Ghent University 0P3, De Pintelaan 185, 9000 Gent

mom_vragenlijst_def_ENG.indd 1 27/05/10 15:26

INSTRUCTIONS FOR COMPLETING THE QUESTIONNAIRE

This questionnaire forms part of a study at Ghent University on **difficult moments and feelings** during pregnancy. It is known that feelings affect pregnancy. This study is conducted in order to understand how your feelings affect your pregnancy and thereby improve health care. We would like to ask you to complete the exact same questionnaire at 20-22 weeks of your pregnancy and at 32-34 weeks. It will take approximately **10 minutes** to complete the questionnaire.

In order to link these two questionnaires and still guarantee your anonymity as much as possible, we kindly ask you to fill out the following code in capital letters: **the first two letters of your first name, the first two letters of your family name, and the month and year of your date of birth**.

Complete:

………. / ………. / ………. / 19 ……………..

For example::
Marie Smith, born in August 1978 becomes: ***MA*** / ***SM*** / ***08*** / 19***78***

Not just the pregnancy, but also the period after childbirth can be accompanied by difficult moments and conflicting feelings. That is why we would like to study the experiences and emotions of a number of women who completed the questionnaires during their pregnancy **at two different moments after childbirth**. If you are willing to help us out with this, we will contact you for two telephone interviews, one month and five months after childbirth. During these interviews, we intend to map your experiences and feelings in more detail and explore ways to take better care of women. The interviews will take approximately **one hour**. Your participation is essential to improve perinatal care! Therefore we will offer you a **gift voucher of 20€** if you are willing to be interviewed twice. We would like you to fill out your data below in capital letters, so that we are able to contact you.

ALL INFORMATION FROM THIS QUESTIONNAIRE WILL BE TREATED AS STRICTLY CONFIDENTIAL. YOUR GYNAECOLOGIST, MIDWIFE OR ANYONE ELSE WILL NOT BE INFORMED OF THE CONTENTS OF THIS QUESTIONNAIRE.

Name and familyname: ……………………………………………………………………………………………

Land line/cell phone no.: ……………………………………………………………………………………………

E-mail: ……………………………………………………………………………………………

How do you proceed?

STEP 1: Read the information guide on pages 11 & 12 and sign the Informed Consent Form on page 13 ;
STEP 2: Complete the entire questionnaire;
STEP 3: Tear off the final page: you can take the blue copy of the Informed Consent Form [page 15] home as proof of your participation in this study;
STEP 4: Put the questionnaire in the envelope provided and seal it;
STEP 5: Put the envelope in the box.

THANK YOU IN ADVANCE FOR YOUR PARTICIPATION IN THIS STUDY!

QUESTIONNAIRE

As this questionnaire will be scanned in, it is important to pay strict attention to the following elements:

- Completely colour the option that is applicable to you as follows ' ● ' . In some questions, it is possible to colour multiple options;
- If you made a mistake, cross out the incorrect answer as follows: ' ☒ ' and then colour the correct one;
- Please write clearly when filling out a text or a number.

1. **Date at which this questionnaire was completed:** .... / .... / 20 ....
2. **How many weeks are you pregnant at this moment:** .... weeks
3. **How old are you?** .... years old
4. **What is your marital status?**

O Married O Living together O Divorced or Separated O Single

5. **What is the highest certificate or degree that you have achieved?**

O None O Non-university higher education

O Primary education O University higher education

O Secondary education

6. **How did you feel most of the time DURING THE PAST 2 WEEKS?**
There are no 'right' or 'wrong' answers, colour the option that reflects best how YOU felt during the past 2 weeks.

	Never	Rarely	Sometimes	Often	Almost always
a. **I feel pleasant**	O	O	O	O	O
b. **In general, I tend to be very tense and nervous**	O	O	O	O	O
c. **I feel lonely**	O	O	O	O	O
d. **I am happy**	O	O	O	O	O
e. **I have crying spells**	O	O	O	O	O
f. **I feel secure**	O	O	O	O	O

	Never	Rarely	Sometimes	Often	Almost always
g. I have difficulty 'shaking off the blues' even with help from my family or friends	O	O	O	O	O
h. All in all, I am inclined to feel that I am a failure	O	O	O	O	O
i. I am calm, cool and collected	O	O	O	O	O
j. Unimportant thoughts often run through my mind and bother me	O	O	O	O	O
k. I feel fearful	O	O	O	O	O
l. I take disappointments so keenly that I can not put them out of my mind	O	O	O	O	O
m. There is a great amount of nervous strain connected with my daily activities	O	O	O	O	O
n. I feel hopeful about the future	O	O	O	O	O
o. I feel depressed	O	O	O	O	O
p. I feel sad	O	O	O	O	O
q. I am content (satisfied)	O	O	O	O	O
r. I take a positive attitude toward myself	O	O	O	O	O
s. I lack self-confidence	O	O	O	O	O

7. How did you feel most of the time DURING THE PAST 2 WEEKS?

There are no 'right' or 'wrong' answers, colour the option that reflects best how YOU felt during the past 2 weeks.

	Strongly disagree	Somewhat disagree	Undecided	Somewhat agree	Strongly agree
a. I am easily bothered by things that did not use to bother me	O	O	O	O	O
b. I have little control over the things that happen to me	O	O	O	O	O
c. There is little I can do to change many of the important things in my life	O	O	O	O	O

mom_vragenlijst_def_ENG.indd 4 27/05/10 15:26

	Strongly disagree	Somewhat disagree	Undecided	Somewhat agree	Strongly agree
d. I feel that I do not have much to be proud of	O	O	O	O	O
e. I am able to do things as well as most other people	O	O	O	O	O
f. I often feel helpless in dealing with the problems of life	O	O	O	O	O
g. I feel that I have a number of good qualities	O	O	O	O	O
h. I feel that I am a person of worth, at least on an equal plane with other people	O	O	O	O	O
i. There is really no way I can solve some of the problems I have	O	O	O	O	O

8. Have you ever been emotionally or physically abused by your partner or someone important to you?

O Yes O No

9. DURING THE 12 MONTHS PRIOR TO YOUR PREGNANCY, were you hit, slapped, kicked or otherwise physically hurt by someone?

O Yes O No → go to question **10.**

↓

a. **Who?** Colour all options that apply.

O Spouse O Partner O Family member

O Ex-spouse O Ex-partner O Stranger

O Other

b. **How often?** [during the 12 months prior to your pregnancy]

O Rarely O Occasionally O Often O Very often

10. SINCE YOU BECAME PREGNANT, have you been hit, slapped, kicked or otherwise physically hurt by someone?

O Yes O No → go to question **11.**

↓

a. **Who?** Colour all options that apply.

- O Spouse
- O Partner
- O Family member
- O Ex-spouse
- O Ex-partner
- O Stranger
- O Other

b. **How often?** [during the 12 months prior to your pregnancy]

- O Rarely
- O Occasionally
- O Often
- O Very often

c. **In terms of its severity and/or frequency, has this behaviour:**

- O Increased
- O Decreased
- O Remained unchanged

11. DURING THE 12 MONTHS PRIOR TO YOUR PREGNANCY, did anyone force you to have sexual activities?

- O Yes ↓
- O No → go to question 12.

a. **Who?** Colour all options that apply.

- O Spouse
- O Partner
- O Family member
- O Ex-spouse
- O Ex-partner
- O Stranger
- O Other

b. **How often?** [since you became pregnant]

- O Rarely
- O Occasionally
- O Often
- O Very often

12. SINCE YOU BECAME PREGNANT, has anyone forced you to have sexual activities?

- O Yes ↓
- O No → go to question 13.

a. **Who?** Colour all options that apply.

O Spouse　O Partner　O Family member

O Ex-spouse　O Ex-partner　O Stranger

O Other

b. **How often?** [since you became pregnant]

O Rarely　O Occasionally　O Often　O Very often

c. **In terms of its severity and/or frequency, has this behaviour:**

O Increased　O Decreased　O Remained unchanged

13. **Are you afraid of your partner or anyone you listed above?**

O Yes　O No

14. If you did not have a partner in the 12 months prior to your pregnancy, nor since you became pregnant → go to question 16.
When you think about your current or last partner, did he/she in the 12 MONTHS PRIOR TO YOUR PREGNANCY:

a. **Try to restrict your contact with male/female friends and/or family?**

O Never　O Rarely　O Occasionally　O Often　O Very often

b. **Insist on knowing where you are at all times?**

O Never　O Rarely　O Occasionally　O Often　O Very often

c. **Ignore you and treat you indifferently?**

O Never　O Rarely　O Occasionally　O Often　O Very often

d. **Insult you, criticize you, or react in a despising manner to what you do or say?**

O Never　O Rarely　O Occasionally　O Often　O Very often

mom_vragenlijst_def_ENG.indd 7　27/05/10 15:26

e. **Belittle or humiliate you in front of other people?**

O Never O Rarely O Occasionally O Often O Very often

f. **Do things to scare or intimidate you on purpose?** [e.g. smashing things, threatening to kill you or to commit suicide]

O Never O Rarely O Occasionally O Often O Very often

g. **Threaten to hurt you or someone you care about?**

O Never O Rarely O Occasionally O Often O Very often

15. **When you think about your current or last partner, has he/she SINCE YOU BECAME PREGNANT:**

a. **Tried to restrict your contact with male/female friends and/or family?**

O Never O Rarely O Occasionally O Often O Very often

b. **Insisted on knowing where you are at all times?**

O Never O Rarely O Occasionally O Often O Very often

c. **Ignored you and treated you indifferently?**

O Never O Rarely O Occasionally O Often O Very often

d. **Insulted you, criticized you, or reacted in a despising manner to what you do or say?**

O Never O Rarely O Occasionally O Often O Very often

e. **Belittled or humiliated you in front of other people?**

O Never O Rarely O Occasionally O Often O Very often

f. **Done things to scare or intimidate you on purpose?** [e.g. smashing things, threatening to kill you or to commit suicide]

O Never O Rarely O Occasionally O Often O Very often

g. **Threatened to hurt you or someone you care about?**

O Never O Rarely O Occasionally O Often O Very often

h. **If you answered any of the above questions from 'a' to 'g' with 'Rarely', 'Occasionally', 'Often' or 'very often', has this behavior SINCE THE BEGINNING OF YOUR PREGNANCY, in terms of severity and/or frequency:**

O Increased O Decreased O Remained unchanged

16. **Did someone else than your current or last partner, behave in more than one of the abovementioned ways? (see '15.a' to '15.g')**

O Yes O No → go to question **17.**

a. **Who?** Colour all options that apply.

O Family member O Stranger O Other

b. **When?**

O during the 12 months prior to your pregnancy

O since you became pregnant

O both during the 12 months prior to your pregnancy and since you became pregnant

17. **How would you rate the CARE provided by your MIDWIFE in the following areas?** [If you did not receive any care from a midwife, colour the option 'Not applicable'. If you have been treated by several midwives during your pregnancy, we are referring to the midwife of your last prenatal check-up]

	Insufficient	Sufficient	Good	Excellent	Not applicable
a. **Provides you with good health care overall**	O	O	O	O	O
b. **Is knowledgeable and competent to follow up on your pregnancy**	O	O	O	O	O
c. **Personally spends enough time with you**	O	O	O	O	O
d. **Makes a special effort to get you to explain your symptoms and complaints completely**	O	O	O	O	O

	Insufficient	Sufficient	Good	Excellent	Not applicable
**e.** **Answers your questions honestly and completely**	O	O	O	O	O
**f.** **Makes sure you understand what you are being told**	O	O	O	O	O

18. How would you rate the CARE provided by your GYNAECOLOGIST in the following areas? [If you did not receive any care from a gynaecologist, colour the option 'Not applicable'. If you have been treated by several gynaecologists during your pregnancy, we are referring to the gynaecologist of your last prenatal check-up]

	Insufficient	Sufficient	Good	Excellent	Not applicable
**a.** **Provides you with good health care overall**	O	O	O	O	O
**b.** **Is knowledgeable and competent to follow up on your pregnancy**	O	O	O	O	O
**c.** **Personally spends enough time with you**	O	O	O	O	O
**d.** **Makes a special effort to get you to explain your symptoms and complaints completely**	O	O	O	O	O
**e.** **Answers your questions honestly and completely**	O	O	O	O	O
**f.** **Makes sure you understand what you are being told**	O	O	O	O	O

THANK YOU VERY MUCH FOR YOUR PARTICIPATION IN THIS STUDY! IF YOU HAVE ANY FURTHER QUESTIONS, PLEASE DO NOT HESTITATE TO CONTACT

An-Sofie Van Parys
Women's Clinic, Ghent University 0P3, De Pintelaan 185, 9000 Gent
Tel: 09/332.53.72 (direct line)
Email: ansofie.vanparys@ugent.be

DO NOT FORGET TO SIGN PAGE 13 & 15

INFORMATION GUIDE FOR PARTICIPANTS

1. **Study title:**
   Difficult moments and feelings during pregnancy.

2. **Aims of the study:**
   This questionnaire forms part of a study at the Ghent University on difficult moments and feelings during pregnancy. It is known that feelings have an impact on pregnancy. This study is conducted in order to understand how your feelings affect you pregnancy and to provide better care.

3. **Description of the study:**
   Please turn to the “Instructions for completing the questionnaire” on the page 2.

4. **What is expected of the participant?**
   Your full cooperation with the researcher and strict compliance with her instructions are extremely relevant for the successful completion of the study. We would like to ask you to complete a short questionnaire on the spot at two different times (between the 20th - 22nd and 32nd - 34th weeks of your pregnancy). If you are willing to participate in the second part of this study, you may also be phoned twice for an interview. This study is not intended to provide care. Your gynaecologist, midwife or anyone else will not be informed of the contents of your questionnaire.

   If you would nonetheless like to talk to someone or require support, feel free to contact:
   - “Tele-onthaal” (Support Hotline): dial 106 or 107;
   - The prenatal care providers (gynaecologist, midwife, nurse) and the social services of the hospital.

5. **Participation and termination:**
   Any participation in this study is voluntary and cannot be considered as providing an immediate therapeutic advantage. Your participation may contribute to better care for women who are pregnant or who have only just delivered. You have the right to refuse to participate and can withdraw from the study at any time, without needing to provide an explanation and without any consequences for your continued relationship and/treatment with the researcher or doctor in charge. Your participation in this study will be terminated if the researcher deems this to be in your interest. You can also be withdrawn prematurely from this study if you decide not to follow the procedures properly or if you fail to respect the items described. If you do decide to participate, you are requested to sign the Informed Consent Form.

6. **Procedures:**

   6.1. Procedure:
   You will be briefed on this study by a care provider or a receptionist at the hospital where go for your prenatal care. If you are willing to participate, you will receive an envelope with this informative guide (including the informed consent form) and the questionnaire. If you remain interested after having read this, please sign the Informed Consent Form.

   As mentioned before, you will be requested to complete a short **questionnaire** in the hospital at the different phases of your pregnancy (once between 20th - 24th week and once between the 32nd - 34th week). Please deposit the completed questionnaire and the signed Informed Consent Form in a sealed envelope in the box provided. You may keep a copy of the Informed Consent Form at the end of this package as proof of your participation.

   If you are selected to participate in the second part of the study, you will receive an envelope from the researcher during the first prenatal consultation after the second questionnaire. The researcher will then contact you for a **telephone interview** at approximately one month and five months after your delivery. We can offer you a **gift voucher of 20€** if you are willing to be interviewed twice on the telephone.

6.2. **Research proceedings:**

Phase 1: Questionnaire between the 20th - 22nd week and between the 32nd -34th week;

Phase 2: The envelope is handed over to you;

Interview 1 and 5 months after your delivery.

7. **Risks and advantages:**

You have the right to enquire about the possible and/or known risks and disadvantages of this study at all times. You will be informed if any data might be revealed in the course of this study that could affect your willingness to continue to participate. You will receive appropriate treatment should you still experience any repercussions as a result of your contribution. This study has been approved by an independent Medical Ethics Commission at Ghent University Hospital and will be carried out according to the international guidelines for good clinical practice (ICH/GCP) and the Helsinki Declaration for the protection of participations in clinical research. Under no circumstances should you consider the approval by the Medical Ethics Commission as an incentive to participate.

8. **Costs:**

Your participation in this study carries no extra costs for you.

9. **Compensation:**

If you are selected and willing to be interviewed, you will receive a gift voucher with a value of 20€.

10. **Confidentiality:**

In compliance with the Belgian laws of the 8th of December 1992 and the 22nd of August 2002, your right to privacy will be respected and you will be granted access to all the gathered data. Any erroneous information can be corrected at your request. Direct access to the files is granted to representatives of the organization commissioning this research project, auditors, the Medical Ethics Committee and authorized governmental agencies, in order to verify the research procedures and/or the data, without infringing your confidentiality. This is only permitted within the boundaries that are defined by applicable law. You are expressing your consent with this procedure by signing the Informed Consent Form after a short briefing. If you agree to participate in this study, your personal and medical data will be collected and coded (in this process it will still be possible to link that information to your personal file). Any reports in which you are identified will not be publicly available. Your identity will remain confidential if the study's results are published.

11. **Injury as a result of participation in this study:**

The researchers foresee a compensation and/or medical treatment should you suffer any harm and/or injury as a result of participation in this study. A zero liability insurance policy has been signed for that purpose in compliance with the law regarding experiments with human test subjects of the 7th of May 2004. In this event, your data will be passed on to the insurance company.

12. **Contacts:**

Feel free to contact the following persons at any time during the study should you experience any harm or if you would like to receive additional information regarding the research project or your rights and duties:

An-Sofie Van Parys
Midwife, sexologist and PhD student,
Women's Clinic, Ghent University
Tel: 09/332.53.72
Email: ansofie.vanparys@ugent.be

Dr. Hans Verstraelen
M.D. and scientific associate,
Women's Clinic, Ghent University
Tel: 09/332.37.96

Prof. dr. Marleen Temmerman
Gynaecologist and Head of Department,
Women's Clinic, Ghent University
Tel: 09/332.37.96

mom_vragenlijst_def_ENG.indd 12 27/05/10 15:26

INFORMED CONSENT FORM

I, ... hereby declare that I have read the "Information guide for participants in experiments" including pages 11 and 12 and that I have received a copy of this document (see page 15). I agree with its contents and also agree to participate in this study.

I have received a copy of this signed and dated "Informed Consent Form". I have been briefed on the nature, the aim, the length and the foreseen effects of this study and on what is expected of me. The possible risks and advantages of the study have been explained to me. I have had the opportunity and sufficient time to ask questions about this study, and I have received a satisfactory answer to all my questions.

I hereby agree to fully cooperate with the supervising researcher. I will inform him/her if I experience any unexpected or unusual symptoms.

I have been informed about the existence of a liability insurance policy should I suffer any harm that can be ascribed to the research procedures.

I am aware that this study has been approved by an independent Medical Ethics Commission at the Ghent University Hospital and that it will be conducted according to the guidelines for good clinical practice (ICH/GCP) and the Declaration of Helsinki for the protection of human research subjects. However, this approval did not influence my decision in any way to participate in this study.

I can withdraw from the study at any time without having to provide a reason for this decision and without jeopardizing my continued relationship with the researcher.

I have been duly informed that both my personal data and the data related to my health, race and sex life will be processed and stored for at least 20 years. I consent to this process and am aware that I have the right to access these data. I understand that my access to these data can be postponed until after the study has been completed, since these data are processed for medical-scientific purposes. I will contact the supervising researcher responsible for processing should I wish to access my data.

I understand that auditors, representatives of the organization commissioning the researcher, or authorized governmental agencies might want to inspect my data to inspect the gathered information. I give my permission for such inspections, even if this means that my data might be passed on to a country outside the European Union. My privacy will still be respected at all times.

I am willing to take part in this study on a voluntary basis

Name of the person ....

Date: ....

Signature: ....

I have explained the nature, the aim and the predicted effects of the study to the abovementioned volunteer.
The volunteer agreed to take part by signing with his/her personal and duly dated autograph.

Name of the person who provided the explanation ....

Date: ....

Signature: ....

mom_vragenlijst_def_ENG.indd 13 27/05/10 15:26

An-Sofie Van Parys
Midwife, sexologist and PhD student
Women's Clinic, Ghent University 0P3, De Pintelaan 185, 9000 Gent
Tel: 09/332.53.72 (direct line)
Email: ansofie.vanparys@ugent.be

mom

UNIVERSITEIT GENT

COPY INFORMED CONSENT FORM

I, ... hereby declare that I have read the "Information guide for participants in experiments" including pages 11 and 12 and that I have received a copy of this document (see page 15). I agree with its contents and also agree to participate in this study.

I have received a copy of this signed and dated "Informed Consent Form". I have been briefed on the nature, the aim, the length and the foreseen effects of this study and on what is expected of me. The possible risks and advantages of the study have been explained to me. I have had the opportunity and sufficient time to ask questions about this study, and I have received a satisfactory answer to all my questions.

I hereby agree to fully cooperate with the supervising researcher. I will inform him/her if I experience any unexpected or unusual symptoms.

I have been informed about the existence of a liability insurance policy should I suffer any harm that can be ascribed to the research procedures.

I am aware that this study has been approved by an independent Medical Ethics Commission at the Ghent University Hospital and that it will be conducted according to the guidelines for good clinical practice (ICH/GCP) and the Declaration of Helsinki for the protection of human research subjects. However, this approval did not influence my decision in any way to participate in this study.

I can withdraw from the study at any time without having to provide a reason for this decision and without jeopardizing my continued relationship with the researcher.
I have been duly informed that both my personal data and the data related to my health, race and sex life will be processed and stored for at least 20 years. I consent to this process and am aware that I have the right to access these data. I understand that my access to these data can be postponed until after the study has been completed, since these data are processed for medical-scientific purposes. I will contact the supervising researcher responsible for processing should I wish to access my data.

I understand that auditors, representatives of the organization commissioning the researcher, or authorized governmental agencies might want to inspect my data to inspect the gathered information. I give my permission for such inspections, even if this means that my data might be passed on to a country outside the European Union. My privacy will still be respected at all times.

I am willing to take part in this study on a voluntary basis.

The volunteer's name: ....

Date: ....

Signature: ....

I have explained the nature, the aim and the predicted effects of the study to the abovementioned volunteer.
The volunteer agreed to take part by signing with his/her personal and duly dated autograph.

Name of the person who provided the explanation: ....

Date: ....

Signature: ....

mom_vragenlijst_def_ENG.indd 15 27/05/10 15:26

An-Sofie Van Parys
Midwife, sexologist and PhD student
Women's Clinic, Ghent University 0P3, De Pintelaan 185, 9000 Gent
Tel: 09/332.53.72 (direct line)
Email: ansofie.vanparys@ugent.be

mom_vragenlijst_def_ENG.indd 16 27/05/10 15:26
